# Role of Salivary Biomarkers in Cystic Fibrosis: A Systematic Review

**DOI:** 10.1155/2022/5818840

**Published:** 2022-01-19

**Authors:** Asma Almeslet, Sarah Alnamlah, Latifa Alanzan, Rawan Aldriwesh, Sha'eah AlWehaiby

**Affiliations:** ^1^Assistant professor, Department of Oral Maxillofacial Surgery and Diagnostic Sciences, Riyadh Elm University, Riyadh, Saudi Arabia; ^2^Faculty of Dentistry, King Saud University, Riyadh, Saudi Arabia; ^3^Faculty of Dentistry, Princess Nourah Bint Abdulrahman University, Riyadh, Saudi Arabia; ^4^Faculty of Dentistry, Majmaah University, Zulfi, Saudi Arabia; ^5^College of Dentistry, Dar Al Uloom University, Riyadh, Saudi Arabia

## Abstract

**Background:**

Saliva biomarkers could be easily used as a noninvasive alternative tool for diagnosing cystic fibrosis (CF) disease. In this study, the significance of changes in salivary compositions in patients with CF was systematically reviewed.

**Methods:**

An electronic search was utilized to include studies published in English, with case-control, cohort, or cross-sectional design. The evaluated salivary components were extracted and summarized. The included studies were assessed using the Strengthening the Reporting of Observational Studies in Epidemiology checklist.

**Results:**

Out of 498 identified studies, nine met the eligibility criteria. Salivary electrolytes showed a substantial alteration in the CF group, especially with chloride and sodium. Total protein concentration was higher in patients with CF. However, SCN– concentration was lower in patients with CF. In addition, a reduction in the salivary flow rate and amylase levels was found in patients with CF.

**Conclusion:**

Alterations in salivary biomarkers among patients with CF could be used as a promising diagnostic tool for cystic fibrosis.

## 1. Introduction

Cystic fibrosis (CF) is a life-limiting, multisystem autosomal recessive genetic disorder with a wide range of clinical and genetic variants [1]. CF most commonly affects Caucasians, with 70,000 people diagnosed worldwide [2]. It is caused by gene mutations in the CF transmembrane conductance regulator (CFTR) on the long arm of chromosome 7 that contributes to an abnormal chloride and sodium transportation across the epithelial cell membrane. As a result, this alteration affects hydration and mucociliary transport within exocrine glands, including the salivary glands [3]. CF is usually diagnosed on the basis of evidence of CFTR dysfunction, which is based on an abnormal sweat chloride test or the CFTR gene mutation. Other diagnostic tests may include immunoreactive trypsinogen test, sputum test, chest X-ray, CT scan, or pulmonary function tests.

Monitoring of CF has included sampling of numerous biofluids. In addition to the genetic test of CFTR mutations, the gold standard diagnostic method is chloride ion concentration (≥60 mEq/mL in sweat) [4]. Saliva was later introduced as a diagnostic modality [5]. Saliva has been utilized as a diagnostic tool for oral and systemic diseases [6–9]. Its use as an early detection approach has attracted special attention. It has been highly recognized due to its noninvasive accessibility, easy performance by modestly trained individuals, and simple equipment that could be used to collect salivary samples. Offering a cost-effective solution for screening larger population is considered the advantage of saliva over serum.

CF respiratory disease has been selected to confirm saliva's diagnostic technique based on well-founded studies of sputum and blood inflammation markers. Furthermore, many of these publications reported significant differences in the levels of different protein markers among patients with CF and healthy subjects [5]. In addition, salivary electrolytes have exhibited some changes depending on various CF-related factors [6].

Upon careful search in different databases about salivary biomarkers and their association with CF disorder, few studies investigated the changes in salivary components and biomarkers in patients with CF. Therefore, the present study is aimed at systematically reviewing the significance and medical uses of the changes in the salivary composition of patients with CF and evaluating the feasibility of using these biomarkers for diagnosis and clinical assessment of CF disorder.

## 2. Materials and Methods

### 2.1. Search Strategy and Selection of Studies

The search strategy was planned in accordance with the guidelines of Preferred Reporting Items for Systematic Reviews and Meta-Analyses [10]. The review of literature was based on the research question “what are the substantial changes that occurred in the saliva of patients affected by CF” and developed using Patient, Intervention, Comparison, and Outcome format [10]. This review covered published studies in English from the interval period of January 2000–December 2019. Observational studies, including case-control, cohort, or cross-sectional studies, concerning the question of this systematic review were included for analysis. PubMed, Scopus, Web of Science, EBSCO, and Cochrane Library were searched. The search was accomplished through the indexation of MeSH by using various combinations of terms, including “cystic fibrosis, saliva, saliva biomarkers, salivary enzymes,” with prefixes “AND” and “OR” to involve all the relevant studies in the particular specified time of publication. Moreover, the reference lists of the included studies were manually searched for any additional relevant articles.

### 2.2. Data Extraction and Quality Assessment

The following data were measured: author and year, type of study, CF group (number of participants and age), control groups (number of participants and age), measured saliva parameters, and primary outcomes of patients with CF compared with those of the control group from each included study were extracted, analyzed in detail, and then summarized in a table. In addition, quality assessment of the included studies was carried out to restrict the risk of bias by using the Strengthening the Reporting of Observational Studies in Epidemiology checklist and graded in accordance with the Olmos scale [11–13] as follows: A, if the study fulfilled >80% of the STROBE criteria; B, if 50%–80% of the criteria were met; and C, if <50% of the criteria were met.

## 3. Results

**Figure 1:**
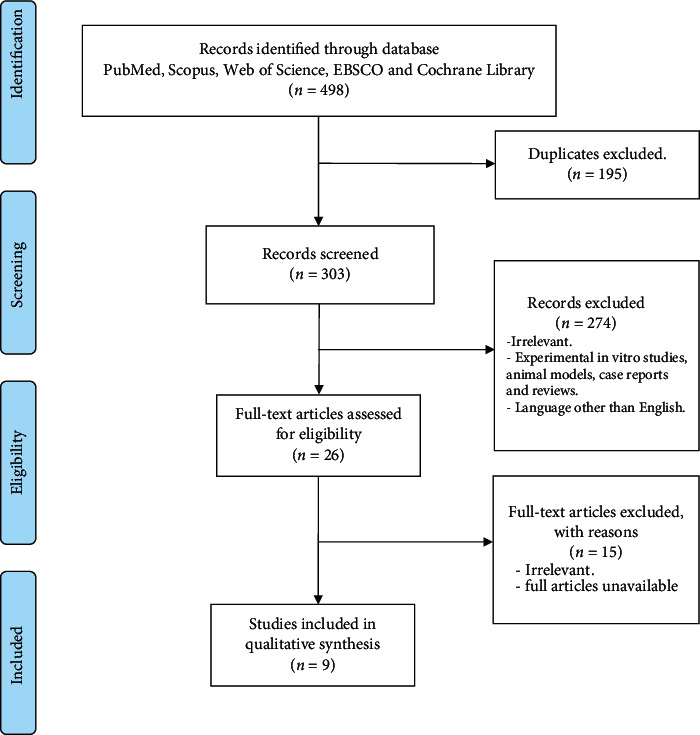
PRISMA flowchart of the study selection process.

A total of 498 articles were retrieved from the search databases. After duplicates were excluded, 303 articles were analyzed. On the basis of the information provided in the study title and abstract, 274 publications were excluded for the following reasons: (1) irrelevant to the focus of this systematic review; (2) different language other than English; or (3) experimental in vitro studies, animal models, case reports, and reviews. The full texts of the remaining 24 articles were retrieved and screened for eligibility. Nine publications met the eligibility criteria, and they were included in this systematic review (Figure 1).

### 3.1. Study Characteristics

The characteristics of the included studies are summarized in Table 1. Of the nine included studies, six had comparable participants in the experimental group (patients with CF) and the control group [14–19]. However, the participants in the control group were significantly higher than patients with CF in one study [20]. By contrast, patients with CF were higher than the control group in one study [5], but this characteristic was unclear in the study by Minarowski et al. [21]. The mean age of participants was comparable in patients with CF compared with the control group in seven studies [5, 14–18, 20], while the mean age was not reported in two studies [4, 21]. Male and female participants were comparable in six studies [5, 15, 16, 18–20]. However, the number of females was significantly higher than that of males in the control group in one study [17], and no information was reported regarding males and females in two studies [14, 21].

The concentration of the salivary parameters of patients with CF was measured and compared to the salivary parameters of the healthy controls in all included studies. A diversity of the measured salivary parameters, methods of measurements and analysis, and purpose and outcome of each salivary parameter was observed. Moreover, the saliva collection methods were different among the included studies. Aps et al. [14] investigated the heterozygote and homozygote patients with CF to explore the effect of genetic heterogenicity on the salivary components. Minarowski et al. [21] included healthy smokers in the control group to study salivary thiocyanate (SCN–) levels and compared them with patients with CF and healthy nonsmokers. Patients with non-CF bronchiectasis were included as a control group in the study by Livnat et al. [15]. Malkovskiy et al. [19] investigated the levels of SCN– in patients with CF and included those undergoing treatment with CFTR modulators and reported their responses to therapy.

### 3.2. Quality Assessment

Among the included studies, one was graded A score [5], five studies were graded B score [14–20], and one study was graded C score [21]. Most pitfalls were in the methodology and discussion sections as most studies did not provide adequate information about sample size calculation and sampling method. Some others did not report information about the participants. In some studies, key results, limitations, and generalizability were missing in the discussion section (Table 1).

## 4. Discussion

In this review, the result outcomes and the significance level of each biomarker presented in saliva and the validity of using these biomarkers in the diagnosis, clinical assessment, and monitoring of patients with CF were summarized. These parameters included electrolytes, proteins, acids (pH and buffering capacity), enzymes, antioxidants, salivary osmolarity, and flow rate.

### 4.1. Electrolytes

The electrolyte concentrations in the saliva of patients with CF were analyzed using different assessment methods by four studies [14, 15, 18, 20]. Some studies found a substantial alteration that may aid in CF diagnosis, especially with chloride (Cl) and sodium (Na) [14, 18, 20]. The first investigation of salivary electrolytes of CF heterozygotes was conducted by Aps et al. [14]. Although the researchers found that different genotypes of patients with CF have different electrolyte concentrations, the electrolytes were higher in CF homozygotes, especially those with F508 mutation (the most common mutation in patients with CF) [14]. Elevated Na and Cl were also reported in numerous other excluded studies [22–24], while another study reported an opposite result [25]. Phosphate was also higher in patients with CF in the study by Aps et al. [14], while it was not statistically significant in Livnat et al.'s study [15]. Calcium (Ca) was also not statistically different in three studies [14, 15, 18]. Iron (Fe) and magnesium (Mg) were measured in only one study [15], which reported that Fe was not statistically significant and Mg was lower in patients with CF than in the control groups.

In 2013, Gonçalves et al. concluded that Na and Cl are the most reliable electrolytes to be comprehensively investigated as a possible diagnostic tool, because these two elements presented the highest values and sensitivity among other electrolytes. The researchers also recommended further studies with a larger population. In addition, a simultaneous comparison of the level of Na and Cl in saliva and sweat could provide new insights regarding the diagnostic ability of saliva [18]. The authors conducted another research in 2019 and concluded that saliva chloride (SaCl) concentration and saliva sodium (SaNa) concentration are candidates to be used in CF diagnosis [18, 20]. The researchers found a positive correlation between sweat chloride and SaCl and between SwNa and SaNa [20]. However, in their narrative review, Pedersen [26] concluded that the SaNa levels for CF diagnosis are doubtful to be used when saliva is obtained from the submandibular or parotid gland. Nevertheless, the employment of Na-responsive electrodes as a screening tool for CF has shown some potentiality.

### 4.2. Proteins

Proteins have been analyzed extensively to diagnose many oral and systemic diseases [26–31]. In the present review, analysis of protein concentration in saliva was reported by four studies [5, 15–17]. Total protein concentration was higher in patients with CF in three studies [15–17]. Furthermore, albumin levels and glycoprotein concentration were found to be not statistically significant [15, 16]. Total protein concentration was determined to be higher in saliva samples before salivary stimulation [16]. Salivary inflammatory cytokines were elevated in patients with inflammatory diseases [32]. Such findings encouraged researchers to investigate these salivary proteins in patients with CF by using promising platforms [33, 34]. Another study examined the levels of six proteins (VEGF, IP-10, IL-8, EGF, MMP-9, and IL-1*β*) in two different time points by using two different platforms, and significant elevations in IP-10 and IL-8 were found. Meanwhile, a reduction in MMP-9 was observed in patients with CF compared with the control group. More interestingly, the levels of these proteins were correlated with the clinical assessment of patients with CF and their ability to be used as biomarkers for specific infections. Researchers found a significant correlation of IP-10 levels with FEV1 and disease severity [5]. In general, the reviewed studies in the present systematic review showed that the total proteins in saliva were higher in patients with CF. [15–17] Other studies also reported higher values of proteins and glycoproteins [35, 36]. Cathepsin D activity was assessed and found to be higher in patients with CF before saliva stimulation, while glycoproteins were not statistically significant [16]. Cathepsin D is a proteolytic enzyme, and it becomes abundant in body fluids, including serum and saliva, during physiological wearing out [37, 38]. The cathepsin D in saliva has also been used to diagnose and monitor patients with breast cancer [39]. Moreover, patients with pulmonary fibrosis and inflammation, including those with CF, showed increased levels of cathepsin D [38].

### 4.3. Thiocyanate and Antioxidant

The concentration of thiocyanates (SCN–) in the saliva of patients with CF is of great concern. Thiocyanate has a role in the host defense system as a substrate for lactoperoxidase, one of the antioxidant systems [21]. One study investigated the mean concentration of SCN– in patients with CF, healthy smokers, and health nonsmokers. The results showed that healthy smokers exhibited the highest levels, followed by healthy nonsmokers and patients with CF. [21] Another study used two different methods for thiocyanate (SCN–) concentration assessment in patients with CF. The researchers investigated if SCN– concentration could be used as a biomarker for CFTR function [19]. The results showed a reduction in the salivary thiocyanate SCN– of patients with CF in both techniques. However, the finding was significant only when Raman spectroscopy was used.

Raman is considered a promising tool due to its ability to differentiate patients with CF and CFTR modulators, those with CF but without modulators, and healthy subjects. Furthermore, Raman was used to measure SCN– in a subject with G551D mutation before and after administration of ivacaftor, one of the CFTR modulators. The authors concluded that Raman could be used to assess the CFTR function through salivary thiocyanate concentration [19].

Oxidative stress elevation is considered part of the pathogenesis of CF and other inflammatory diseases. As a consequence of its elevation, many harmful effects have been raised, such as inflammatory injury, losing control over the inflammation process, organ failure, and dysfunction. These effects increase the importance of antioxidants, including the salivary antioxidant system in the oral cavity, for further protection against their harmful effects [4, 40]. A reduction in peroxidase and an elevation in superoxide dismutase activities, uric acid concentration, and total antioxidant status have also been observed in patients with CF. [15] Most of the salivary antioxidant enzymes and molecules were altered in patients with CF. This finding is related to the decrease in the defense against oxidative stress, which may be of clinical importance considering the primary risk of patients with CF. [15] Following another study, a reduction in salivary peroxidase by 55% was observed in patients with CF compared with the control group [17].

### 4.4. Amylase, Lactate Dehydrogenase (LDH), Glucose, Lactate, Bicarbonate, and Sialic Acid

The *α*-amylase digestive enzyme is one of the highly copious components of saliva. It breaks down carbohydrates to help with indigestion. Moreover, it could bind with some oral bacteria and participate in bacterial clearance [41]. A significant reduction in the amylase levels by 55% was found in patients with CF compared with the control group [17]. This reduction of amylase and salivary peroxidase could contribute to undesirable effects in the oral cavity of patients with CF. [42]

Conversely, another study did not record any statistically significant difference in amylase levels [15]. The authors evaluated various other changes in salivary composition, including LDH, which showed a significant decrease by 55% in the saliva of the CF group compared with the healthy control group. This finding could be responsible for the oral mucosal changes in patients with CF. [15] The investigation of sialic acid showed a reduction of its concentrations in saliva (total, free, and conjugated to glycoproteins) of patients with CF. [17] This acid is found in mucin and other glycoproteins; it also plays an essential role in protecting oral mucosa in providing lubrication and maintaining mucosal permeability and preventing the penetration of harmful substances [43]. No significant differences were found in glucose, lactate, and bicarbonate in the saliva of patients with CF. [14, 18]

### 4.5. Salivary Flow Rate, pH, Osmolarity, and Buffering Capacity

Salivary flow rate was measured in three studies [15, 17, 18]. A reduction in the salivary flow rate in patients with CF was observed in two studies [17, 18]. By contrast, Livnat et al. reported that salivary flow rate and pH in patients with CF were similar to those in the healthy control group [15]. However, a large-scale study by Gonçalves et al. [18] reported a reduction of salivary pH in patients with CF. Another aspect of this topic is the buffering capacity of saliva, which is essential for neutralizing and keeping the oral cavity pH; it is also considered critical for dental remineralization and demineralization [17, 18]. da Silva Modesto et al. [17] measured saliva's total pH and buffering capacity. They also measured the buffering capacity in three different ranges of pH (pH > 7, 6.9–6.0, and <5.9). They found no difference in the initial pH and the total buffering capacity in patients with CF compared with the control group; however, a reduction in the buffering capacity was observed in the pH range of 6.9–6.0. Salivary osmolarity was investigated in only one study. It was higher in CF homozygotes due to an increase in the concentration of some organic and inorganic components and/or reduced water content of saliva [14].

*Pseudomonas aeruginosa* in patients with CF and IgA of saliva has been recently investigated for their diagnostic purposes. Sinus colonization could eventually lead to intermittent lung colonization, which proceeds to chronic infection. Sinus colonization results in elevated salivary IgA, specifically against *P. aeruginosa*. It aids in the early detection of bacteria to prevent further progression and lung colonization, which was discussed in several studies [44–47]. This relation initiates further research on salivary IgA and its possible prediction of the changes of lung infection in patients with CF.

A notable detail that the results obtained suggested that salivary biomarkers exhibit changes in CF, indicating their possibility as a diagnostic tool. However, several limitations have been encountered in the included studies as follows: (i) the methods for assessing salivary parameters differ, which hinders comparisons, and (ii) several studies have been performed with small sample size or inappropriate age/gender distribution. Such limitations made it necessary to recommend further research with better quality, larger populations, and randomization. Moreover, all other variables (e.g., gender, age, different genotypes, and experimental conditions, including the characteristics of participants, assessment methods, and environmental factors) must be controlled to confirm the findings of this review, further improve the measurement accuracy of saliva parameters in patients affected by CF, and strengthen the clinical uses of saliva.

## 5. Conclusion

In conclusion, saliva profile is altered due to CF pathogenesis. These alterations contribute to various effects in antimicrobial, antioxidant, lubricating, and digestive functions. Overall, the results emphasized the potential of using salivary biomarkers in the diagnosis, clinical evaluation, and monitoring of patients with CF. In addition, further controlled studies are highly recommended to confirm these findings.

## Figures and Tables

**Table 1:** Characteristics and outcomes of the included studies.

Author, year	Type of study	Quality	Characteristics of participants		Saliva parameters	Outcomes in the CF group compared to the control group	
			CF group	Control group			
Aps et al., 2002 [14]	Case control	B	CF homozygotes *N* = 46 Age = 17.2 ± 7.8 CF heterozygotes *N* = 69 Age = 31.2 ± 15.1	*N* = 64 Age = 20.7 ± 11.3	Chloride	Increased*^β^*	
					Potassium	Increased*^α^*	
					Sodium	Increased*^α^*	
					Phosphate	Increased*^α^*	
					Osmolarity	Increased*^α^*	
					Bicarbonate	Increased*^β^*	
					Calcium	Decreased*^β^*	
Minarowska et al., 2007 [16]	Case control		*N* = 26 (12 F, 14 M) Age = 13.3 ± 5.1	*N* = 28 (15 F, 13 M) Age = 13.5 ± 4.6	Cathepsin D	Increased*^α^*	
					Proteins	Increased*^α^*	
					Glycoprotein	Increased*^β^*	
Minarowski et al., 2008 [21]	Case control	C	Not reported	(i) Healthy nonsmokers (ii) Healthy smokers	Thiocyanate (SCN-)	Decreased*^α^*	
Livnat et al., 2010 [15]	Case control	B	*N* = 22 (9 F, 13 M) Age = 13.9 ± 7.1	Non-CF bronchiectasis *N* = 14 (6 F, 8 M) Age = 12.6 ± 4.2 Healthy *N* = 14 (6 F, 8 M) Age = 13.4 ± 4.8	Salivary flow rates	Reported that it is NS compared with CF patients	
					pH	Exactly the same*^β^*	
					Iron, albumin, potassium	Increased*^β^*	
					Calcium	Decreased*^β^*	
					Magnesium	Decreased*^β^*	
					Lactate dehydrogenase	Decreased*^α^*	
					Total protein	Increased*^β^*	
					*α*-Amylase	Increased*^β^*	
					Oral peroxidase	Decreased*^β^*	
					Superoxide dismutase	Increased*^β^*	
					Uric acid	Increased*^β^*	
					Total antioxidant status	Increased*^β^*	
Gonçalves et al., 2013 [18]	Case control	B	*N* = 80 Age = 13.04 ± 7.27	*N* = 84 Age = 13.56 ± 6.03	Salivary volume and pH	Decreased*^α^*	
					Na, Cl, K	Increased*^α^*	
					Calcium	Decreased*^β^*	
					Glucose, lactate, bicarbonate	Increased*^β^*	
da Silva Modesto et al., 2015 [17]	Case control	B	*N* = 21 (47.6% F, 52.4% M) Age = 9.09 ± 2.14	*N* = 28 (64.3% F, 35.7% M) Age = 9.04 ± 2.08	Salivary flow rate	Decreased*^α^*	
					pH	Increased*^β^*	
					Buffering capacity	Decreased*^α^* only in pH range 6.9–6.0	
					Total protein	Increased*^α^*	
					*α*-Amylase activity	Decreased*^α^*	
					Peroxidase activity	Decreased*^α^*	
					Sialic acid concentration	Decreased*^α^*	
Nie et al., 2015 [5]	Cross-sectional	A	Microarray *N* = 71 (39 F, 32 M) Age = 23 SDReader *N* = 117 (58 F, 59 M) Age = 26	Microarray *N* = 56 (34 F, 22 M) Age = 32 SDReader *N* = 50 (30 F, 20 M) Age = 33		Microarray	SDReader
					VEGF	Increased*^α^*	Increased*^β^*
					IP-10	Increased*^α^*	Increased*^α^*
					IL-8	Increased*^α^*	Increased*^α^*
					EGF	Increased*^α^*	Increased*^β^*
					MMP-9	Decreased*^α^*	Decreased*^α^*
					IL-1*β*	Decreased*^β^*	Decreased*^α^*
Gonçalves et al., 2019 [20]	Case control	B	*N* = 57 (30 F, 27 M) Age = 11.77 ± 6.37	*N* = 103 (54 F, 49 M) Age = 9.54 ± 10.42	Salivary sodium, salivary chloride	Increased*^α^*	
Malkovskiy et al., 2019 [19]	Case control	B	*N* = 25 (13 F, 12 M) (14 with stimulated and 11 with unstimulated saliva) CF patients with CFTR modulators *N* = 11 (4 F, 7 M) (2 with stimulated and 9 with unstimulated saliva)	*N* = 23 (9 F, 6 M, 8 unknown) (11 with stimulated and 12 with unstimulated saliva)	SCN− using colorimetry	Decreased*^β^*	
					SCN− using Raman spectroscopy	Decreased*^α^*	

*^α^* Significant difference; *^β^*nonsignificant difference.

## Data Availability

The data supporting the findings of this review are already included.
